# Relation of quantitative flow ratio with transit time coronary artery bypass graft flow measurement

**DOI:** 10.3389/fcvm.2022.975759

**Published:** 2022-09-06

**Authors:** Cong Chen, Yang Zhao, Wei Li, Kui Zhang, Haiming Dang, Taoshuai Liu, Yue Song, Jubing Zheng, Ran Dong

**Affiliations:** Center of Cardiac Surgery, Beijing Anzhen Hospital, Capital Medical University, Beijing, China

**Keywords:** transit-time flow measurement, coronary artery bypass grafting, fractional flow reserve (FFR), coronary artery disease, quantitative flow ratio (QFR)

## Abstract

**Background:**

Quantitative flow ratio (QFR) is a new functional index to assess the functional significance of coronary stenosis. While whether there is an association between QFR and transit-time flow measurement (TTFM) parameters of the target coronary artery has not been well addressed.

**Methods:**

A total of 89 patients receiving the *in situ* left internal thoracic artery (LITA) grafts to the left anterior descending artery (LAD), and 19 patients undergoing the saphenous vein grafts (SVG) were enrolled in this retrospective study. The QFR value of the LAD was evaluated preoperatively. According to the QFR values, patients with the LITA to the LAD bypass grafts were divided into two groups (group A1: QFR < 0.75, group A2: QFR ≥ 0.75), and SVG patients were divided into two groups (V1 group: QFR < 0.75, V2 group: QFR ≥ 0.75).

**Results:**

In groups A1 and A2, respectively, median graft flow (Qm) was 44 (34) mL/minute and 26.5 (30.0) ml/minute; median pulsatility index (PI) was 2.00 (1.00) and 2.65 (0.90). Significant differences were observed in Qm (*P* = 0.034) and PI (*P* = 0.030). And the correlation coefficients of the TTFM variables with QFR were Qm: *r* = *r* = −0.226, (*P* = 0.036), PI: *r* = 0.265 (*P* = 0.012) among the LITA to LAD population.

**Conclusion:**

TTFM variables, especially the PI, of the LITA *in situ* graft to the LAD during Coronary artery bypass grafting (CABG) are strongly affected by preoperative QFR values. Moreover, in functionally mild coronary stenosis, the chance of competitive flow increases.

## Introduction

Coronary artery bypass grafting (CABG) is one of the main therapeutic strategies for coronary artery stenosis and improving the prognosis of patients with coronary heart disease. However, these grafts may become occluded or narrowed over time, especially with grafting less critical stenosis. In coronary surgery, the choice of the target vessel is particularly important. Traditional coronary surgery is performed only based on anatomical methods such as preoperative coronary angiography (CAG), rather than physiological function assessment. However, researchers have found that CAG, as a way to assess coronary stenosis, only based on the anatomical points of view, can easily overestimate or underestimate coronary artery stenosis (1). Assessment of the functional severity of stenosis in a coronary artery is urgently needed.

Several international studies such as the FFR Versus Angiography for Multivessel Evaluation (FAME) study and deferral of percutaneous coronary intervention (DEFER) study have all verified that utilizing functional assessment of coronary artery through the fractional flow reserve (FFR) to guide percutaneous coronary intervention (PCI) has excellent results (2, 3). European Society of Cardiology (ESC) revascularization guidelines have adopted FFR as the gold standard for assessing the severity of coronary artery stenosis (40–90%) (4). Studies have shown that FFR may simplify CABG procedures and optimize arterial graft patency without any significant impact on venous grafts (5–10). FFR-guided CABG was associated with lower overall death, angina, and myocardial infarction rates compared with angiography-guided CABG (6, 8). A prospective study conducted by Botman’s team showed that in patients with FFR ≥ 0.7, the proportion of graft restenosis within 1 year exceeded 10% (11). Honda and his colleagues reported significant differences in graft flow and pulsatility index among the groups (divided by the cutoff value of 0.75 in FFR) (12). These studies provide a clinical basis for us to carry out FFR-guided CABG studies.

Quantitative flow ratio (QFR) is a novel method for evaluating the functional significance of coronary stenosis by computation of FFR in the vessel based on 3-dimensional angiographic reconstruction and fluid dynamics algorithms (13, 14). The Functional Assessment by Various Flow Reconstructions (FAVOR) pilot study showed promising results for core laboratory-based QFR computation in identifying the presence of functionally significant stenosis in selected patients (13). Its accuracy and consistency based on FFR have been widely verified (15, 16). FAVOR III China indicates fewer myocardial infarctions and ischemia-driven revascularisations in the QFR-guided group than in the angiography-guided group (17). The QFR technique only takes 3.6 min to measure FFR and is suitable for more than 97% of coronary angiography images. Overall, QFR technology has high clinical application prospects. However, only limited evidence exists on the role of preoperative QFR in patients undergoing CABG currently, finding that the QFR-guided surgical coronary revascularization is safe and reduces unnecessary bypass grafting (18).

Therefore, our study aimed to analyze the efficiency of QFR in coronary artery functional stenosis assessment and evaluate the feasibility of performing QFR analysis in guiding CABG by exploring the correlation between QFR and the transit-time flow measurement (TTFM) measurement as well as the graft blood flow pattern.

## Patients and methods

### Study patients

This study retrospectively included 108 patients with coronary heart disease undergoing CABG in the sixth Department of Cardiac Surgery of Beijing Anzhen Hospital from January 2019 to June 2020. It has been approved by the hospital ethics committee.

All patients underwent coronary angiography before surgery, and the QFR value of the anterior descending coronary artery was calculated. According to the preoperative QFR value of the descending artery, the patients who undertook the left internal thoracic artery to the anterior descending artery bypass graft were divided into two groups (group A1: QFR < 0.75, group A2: QFR ≥ 0.75), and patients with saphenous vein to the descending branch bypass were divided into V1 group: QFR < 0.75 and V2 group: QFR ≥ 0.75. *In situ* LITA to LAD and SVG to LAD were used. TTFM was used to evaluate the Qm, PI, and systolic reverse blood flow of each group. In addition, the short-term patency of the graft vessel after bypass graft was followed up. Baseline patient characteristics as well as operative procedure data are shown in Table 1.

**TABLE 1:** Patient characteristics.

Variables	*In situ* LITA-LAD (89)		SVG-LAD (19)	
	Group A1 (79)	Group A2 (10)	Group V1 (15)	GroupV2 (4)
Age, year	61.14 ± 7.93	59.50 ± 5.42	68.80 ± 8.79	58.00 ± 13.64
≥ 65 year	27 (34.18%)	2 (20%)	10 (66.67%)	1 (25%)
Male gender	62 (78.4%)	7 (70%)	9 (60%)	4 (100%)
BMI	25.64 ± 3.26	24.96 ± 2.57	23.97 ± 5.60	27.66 ± 2.50
**Past medical history**				
Hyperlipidemia	45 (56.9%)	6 (60%)	6 (73.3%)	2 (50%)
Hypertension	55 (69.6%)	5 (50%)	9 (60%)	4 (100%)
Diabetes mellitus	37 (46.8%)	6 (60%)	6 (40%)	0 (0)
Chronic kidney disease	1 (1.27%)	0 (0)	0 (0)	0 (0)
Pulmonary disease	1 (1.27%)	0 (0)	0 (0)	0 (0)
Previous stroke	5 (6.3%)	1 (10%)	1 (6.6%)	0 (0)
Carotid artery disease	24 (30.3%)	1 (10%)	5 (33.3%)	2 (50%)
Vascular disease	0 (0)	2 (20%)	1 (6.6%)	0 (0)
Unstable angina pectoris	71 (89.87%)	9 (90%)	14 (93.3%)	4 (100%)
PCI	9 (11.39%)	4 (40%)	2 (13.3%)	1 (25%)
Smoking habit	38 (48.1%)	3 (30%)	9 (60%)	2 (50%)
**Left ventricular**				
Ejection fraction, %	60.96 ± 9.19	58.2 ± 11.75	63.27 ± 4.65	53 ± 9.63
End-diastolic dimension, mm	47.66 ± 4.27	50.4 ± 4.55	46.67 ± 3.44	50.25 ± 8.62
End-systolic dimension, mm	31.18 ± 7.51	34 ± 6.7	30 ± 3.4	35.75 ± 8.92
**Coronary angiography**				
Single vessel disease	2 (2.53%)	1 (10%)	0 (0)	0 (0)
Double vessel disease	16 (20.25%)	2 (20%)	4 (26.67%)	1 (25%)
Triple vessel disease	61 (77.22%)	7 (70%)	11 (73.33%)	3 (75%)
**Intraoperative information**				
Operation time, min	205.25 ± 39.33	199 ± 40.12	208.67 ± 53.97	300 ± 14.14
On-pump CABG	2 (2.53%)	0 (0)	0 (0)	0 (0)
IABP usage	2 (2.53%)	1 (10%)	0 (0)	0 (0)
**Postoperative information**				
Ventricular use time, h	16.67 ± 6.89	19 ± 9.21	17.6 ± 10.53	13 ± 4.55
ICU stay, h	25.09 ± 12.34	23.9 ± 13.88	24.73 ± 15.56	20.75 ± 7.5
Hospital stay, d	11.82 ± 3.27	12.2 ± 2.15	10.53 ± 3.02	9.75 ± 2.63

CABG, coronary artery bypass grafting; LITA, left internal thoracic artery; LAD, left anterior descending artery; SVG, saphenous vein graft.

### Preoperative quantitative flow ratio measurement and surgical strategy

**FIGURE 1:**
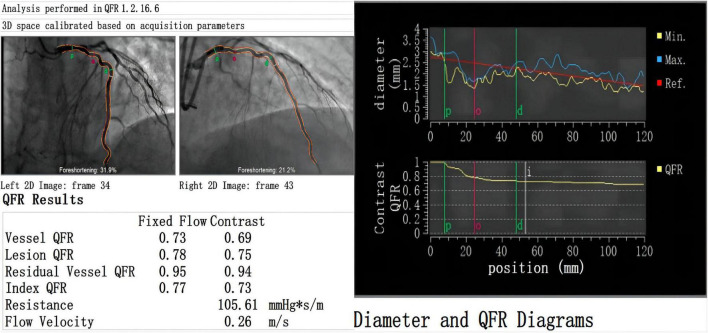
Case example of QFR report. There is a moderate stenosis (diameter stenosis = 46.4%) with QFR value = 0.69 in a 58-year old male patient. Qm (33 ml/min) and PI (3), the backflow component of TTFM is not evident. Postoperative multislice computed tomography angiography shows patency of the LITA graft.

**FIGURE 2:**
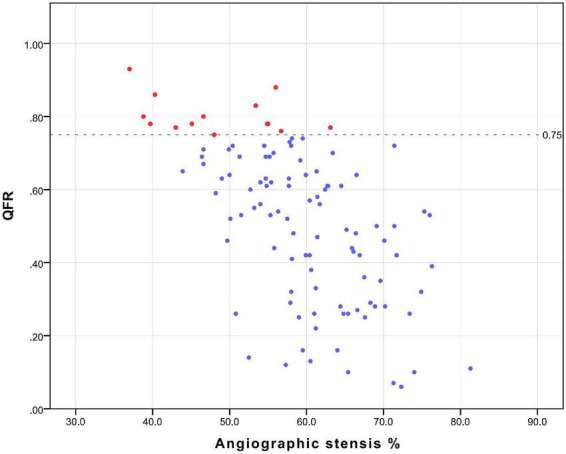
The relation between QFR and the degree of coronary angiographic stenosis.

All patients underwent coronary angiography before surgery, and the results of the angiography were sent to the core laboratory of Shanghai Pulse Medical Technology, Inc., for QFR value calculation and analysis. The results were sent back in reports like that shown in Figure 1. And The relation between QFR and the degree of coronary angiographic stenosis is shown in Figure 2. Cardiac surgeons defined the surgical revascularization strategy based on coronary angiography alone, blinded to the QFR values. Surgery was performed with off-pump CABG (106 cases), on-pump bypass assisted non-stop (1 case), and on-pump CABG (1 case).

### Intraoperative flow measurement

The TTFM was used to measure the graft flow during the operation. The validity of bridge flow measurement is mentioned in the European Society of Cardiology and the European Association of Cardio-thoracic Surgery (ESC-EACTS) guidelines (19). TTFM was measured intraoperatively using a 3 mm or 4 mm flow probe. We routinely obtained TTFM-related parameters twice: (1) just after graft anastomosis during off-pump CABG and on-pump CABG to determine the need for graft modification. (2) In both on-pump and off-pump CABG, when the hemodynamic status becomes stable before chest closure, the mean blood pressure is between 80 and 100 mmHg, the heart rate is between 70 and 100 beats per minute, and a small amount of inotropy and vasopressors used to maintain hemodynamic stability. The parameters measured by the transient-time flow meter include Qm, PI, and systolic reverse flow from the latter flow profiles. Reverse blood flow is measured as the percentage of the area below the basic line, compared with the total flow area. Three months after the operation, patients were evaluated for the patency of the grafts. Multislice computed tomography angiography (MSCTA) was preferred, and individual patients were evaluated by coronary angiography.

## Statistical analysis

STATA 14.0 statistical software was used for data collation and analysis. Categorical variables are expressed as the number of cases (percentage), the chi-square test or Fisher’s exact probability method is used for comparison between groups, and the rank sum test is used for rank variables. Non-normally distributed measurement variables are represented by M (IQR), and the *U* test was used for comparison between groups. Continuous data are presented as mean ± SD. Spearman correlation coefficient was used to evaluate the correlation between preoperative QFR and Qm as well as PI. Two-tailed test, a *P* value less than 0.05 is considered statistically significant.

## Results

A total of 108 patients were divided into 4 groups according to their preoperative QFR value of descending branch. The patients (89 cases) who used LITA-LAD bypass were divided into the A1 group (79 cases): QFR < 0.75, A2 group (10 cases): QFR ≥ 0.75, the patients who used SVG-LAD bypass (19 cases) were divided into V1 group (15 cases): QFR<0.75 andV2 group (4 cases): QFR ≥ 0.75.

Surgery was performed with off-pump CABG (106 cases), on-pump beating-heart (1 case), and on-pump CABG (1 case). All patients underwent coronary artery bypass grafting alone without other cardiac surgery at the same time. There were no deaths or major adverse cardiovascular events during hospitalization.

### Quantitative flow ratio and transit-time flow measurement

**FIGURE 3:**
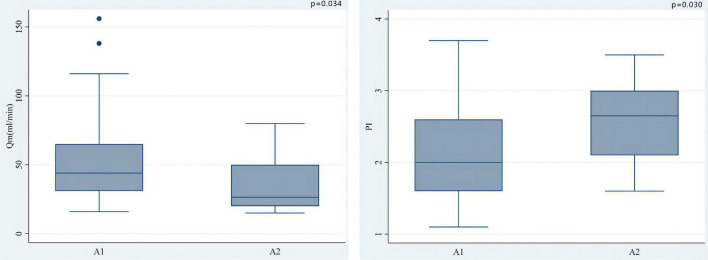
Among patients who used LITA *in situ* graft to LAD, there were significant differences in Qm (*P* = 0.034) and PI (0.030) between the A1 group and the A2 group. *In situ* LITA-LAD graft flow increased with the increase of coronary stenosis severity while the PI decreased.

**FIGURE 4:**
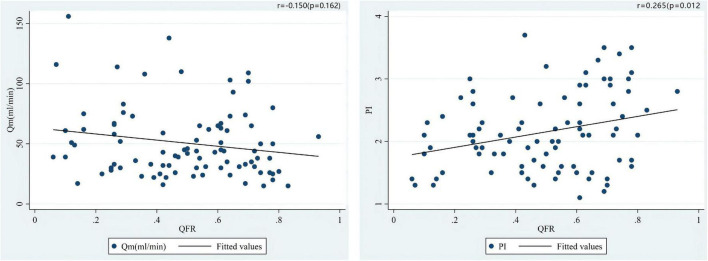
Correlations of the preoperative QFR of the LAD and the parameters of intraoperative TTFM of the *in situ* LITA graft to the LAD, including Qm and PI.

The median of Qm in each group is group A1: 44.0 (34.0)ml/min, group A2: 26.5 (30.0) ml/min, group V1: 67.0 (45.0) ml/min, V2 group: 60.5 (27.3) ml/min; the median of PI in each group were A1 group: 2.0 (1.0), A2 group: 2.7 (0.9), V1 group: 1.4 (0.5), V2 group: 1.8 (0.8); the number of patients with systolic reverse blood flow are 25 (31.65%), 7 (70.00%), 1 (6.25%), and 1 (25.00%) in respective groups. Among patients who used LITA *in situ* graft to LAD, there were significant differences in Qm (*P* = 0.034) and PI (*P* = 0.030) between the A1 group and the A2 group, that is patients with higher QFR have lower Qm and higher PI values (Figure 3). Nevertheless, we did not find a significant difference between the V1 and V2 groups either in PI (*P* = 0.469) or Qm (*P* = 0.596). A negative correlation was observed between QFR and Qm (*r* = −0.150, *P* = 0.162), in contrast, QFR and PI showed positive correlation (*r* = 0.265, *P* = 0.012) (Figure 4). Since the non-significant correlation between QFR and Qm, we carefully analyzed the relevant data and found that EF values were closely correlated with Qm, so we controlled EF values for variables and also dealt with outliers, and the results were satisfactory (*r* = −0.226, *P* = 0.036). And in the young and middle-aged groups (age < 65 years), a more significant correlation (*r* = −0.348, *P* = 0.008) was found. We believe that the insufficient sample size in this study led to such results, and since this is a retrospective trial, its findings are susceptible to bias due to other confounding factors. Therefore, prospective studies with larger sample sizes are needed to investigate the effect of QFR on parameters related to intraoperative grafts.

**FIGURE 5:**
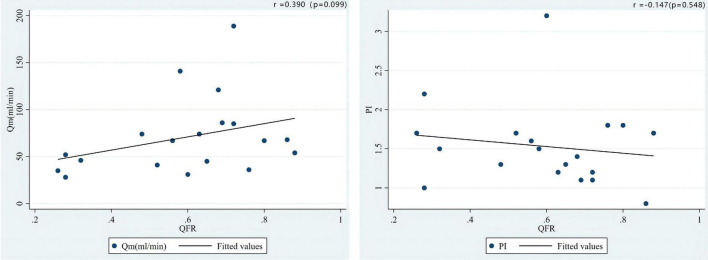
Correlations of the preoperative QFR of the LAD and the parameters of intraoperative TTFM of the SVG to the LAD, including Qm and PI.

**FIGURE 6:**
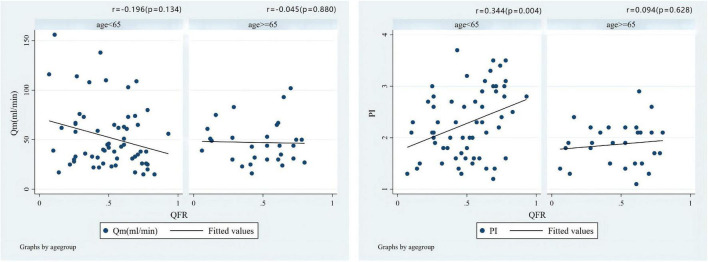
Correlations of the preoperative QFR of the LAD and the parameters of intraoperative TTFM of the *in situ* LITA graft to the LAD, including Qm and PI in different age groups.

As shown in Figure 5, the correlation coefficient between QFR and TTFM-related parameters was *r* = 0.390 (*P* = 0.099) and *r* = −0.147 (*P* = 0.548) for Qm and PI, respectively, in the SVG group which showed an opposite correlation trend when compared with LITA population. Additionally, a positive correlation (*r* = 0.344, *P* = 0.007) between QFR and PI was observed in the LITA group, which was more significant in the young and middle-aged groups (age < 65 years) (Figure 6).

### Patency

The patency of the graft was evaluated in 108 patients (follow-up interval: 139 days). Multislice computed tomography angiography was conducted in 107 patients, and coronary angiography in 1 patient. No death was found in patients.

A distal thread sign at the LITA graft was observed in 1 patient in group A1, and early graft failure in 2 and 1 patient in groups A1 and A2, respectively. Among all the three patients in group A1, Qm and PI showed good performance with no systolic reverse flow observed. MSCTA widely patency rate was 95.5% for the LITA anastomoses, 4 anastomoses were found to be less functional (4.5%); of these, 3 (3.4%) were occluded, 1 (1.1%) presented patent with flow limited. No statistically significant difference was shown between the two groups in terms of patency rate. No patient had symptoms of angina till the latest follow-up.

For the 1 patient identified with early graft failure in group A2, intraoperative Qm was 15 ml/minute, PI was 2.5 and systolic reverse flow was observed. This patient received a stent on the left anterior descending coronary artery which was patent before surgery, and the QFR of the LAD artery was 0.83. Another stent was implanted at the coronary anastomosis 1 year after surgery.

## Discussion

It has become routine to use TTFM for predicting early coronary artery bypass graft failure immediately after surgery (20). The Qm is used to describe the blood flow of the graft, but it was susceptible to many factors, including systolic blood pressure, coronary resistance, graft quality, coronary microcirculation, and anastomotic quality. PI is regarded as an important reference indicator for intraoperative assessment of graft quality. Systolic reverse flow means there is competition for blood flow between the grafts and the native vessels, indicating that blood flow directly refluxes into the graft via anastomosis (21).

Among TTFM parameters, Qm > 15 ml/min, PI < 5.0, and systolic reverse blood flow area < 30% are regarded as indicators of intraoperative graft patency. Especially, Qm and PI, the two important indicators, are most concerned during surgery (22). Mika Noda’s team showed that in LITA-LAD bypass surgery, FFR and most indicators of TTFM are correlated, with a significant negative correlation with Qm, and a significant positive correlation with PI (23). Honda et al. divided LITA-LAD bypass patients into three groups based on the FFR value of the preoperative target vessel and found obvious differences in the Qm and PI among the three groups. As the FFR value increased, the Qm decreased and PI increased subsequently (12). The consistency of QFR and FFR has been verified by many clinical studies (15, 16). Their findings coincide with the results of our study. QFR may be expected to be a more promising index than FFR for the assessment of coronary artery stenosis in the future.

In this study, coronary artery functional stenosis was mainly assessed via QFR, and the relationship between preoperative QFR value and intraoperative TTFM-related parameters, especially Qm and PI, was analyzed. We found that when undergoing *in situ* LITA graft to LAD: (1) Qm and PI are affected by the preoperative QFR value, and with the degree of coronary stenosis increases (that is, a lower QFR value), Qm increases, while PI decreases; (2) In the young and middle-aged population, PI is strongly affected by the preoperative QFR value; (3) The chance of flow competition increases between the native coronary artery and the graft in mild coronary artery stenosis. Moreover, in comparison with that in patients undergoing SVG-LAD bypass graft, the correlation between QFR and TTFM-related parameters such as Qm and PI showed an opposite trend. For saphenous vein grafts, due to the large diameter and the absence of a muscular layer, the pressures at both ends of the circuit are identical, with only slight phasic variations. However, in arterial grafts, the pressure at the proximal port is higher than at the distal anastomosis due to the smaller diameter and higher vasomotor tone. QFR is an assessment of the functional stenosis of the coronary artery, which reflects the hemodynamic situation of the vessel. Since the pressure of the venous grafts is significantly higher than that of the target artery, as the QFR increases, the Qm increases, and the PI decreases. Certainly, this needs to be further studied and validated in a larger sample.

However, there are still some limitations. First, this study is a retrospective study, and the possibility of bias has not been ruled out completely; Second, this study included only a small number of participants who underwent different cardiac operations at a single medical center and more patients should be included for further investigation; Third, we analyzed the correlation in the single arterial graft (*in situ* LITA-LAD) and SVG-LAD patients. Other graft materials such as the right internal thoracic artery, radial artery, gastroepiploic artery, and other arterial materials were not enrolled; Fourth, baseline characteristics affecting LITA such as diameter, calcification, and free flow were not recorded, a detailed assessment could be useful for identifying factors related to QFR and TTFM; Finally, in our study, postoperative follow-up was performed by MSCTA, therefore only the patency of the graft vessel and anastomosis could be observed, but the blood flow direction and competition of the graft vessel were not evaluated. Thus, a more valid evaluation system should be considered in further study to confirm the feasibility of QFR as clinical surgery guidance.

## Conclusion

During *in situ* LITA-LAD CABG, both Qm and PI are greatly affected by QFR. In mild coronary artery stenosis assessed by QFR, the chance of flow competition between the native coronary artery and the bypass graft increased. QFR-guided CABG may be clinically feasible and safe, making it possible to be widely used for clinical surgical guidance.

## Data availability statement

The raw data supporting the conclusions of this article will be made available by the authors, without undue reservation.

## Ethics statement

The studies involving human participants were reviewed and approved by the Ethics Committee of Beijing Anzhen Hospital, Capital Medical University. The patients/participants provided their written informed consent to participate in this study.

## Author contributions

RD, CC, and YZ contributed to conception and design of the study. WL, HD, TL, YS, JZ, and KZ established the database. CC, WL, and YZ performed the statistical analysis and wrote sections of the manuscript. CC wrote the first draft of the manuscript. All authors contributed to manuscript revision, read, and approved the submitted version.
